# Wastewater Treatments Based on Adsorption, Catalysis, Biodegradation, and Beyond

**DOI:** 10.3390/molecules29225470

**Published:** 2024-11-20

**Authors:** Dimitrios A. Giannakoudakis, Yongchang Sun

**Affiliations:** 1 Institute of Chemical Sciences, Faculty of Chemistry, Maria Curie-Sklodowska University, Maria Curie Sklodowska Sq. 3, 20-031 Lublin, Poland; 2 School of Chemistry, Aristotle University of Thessaloniki, 54124 Thessaloniki, Greece; 3 School of Water and Environment, Chang’an University, Xi’an 710054, China

The ongoing technological advancements and industrial growth over the past few decades have resulted in significant environmental challenges, with one of the most notable being water pollution caused by the improper disposal of organic and inorganic pollutants. Protecting the environment is an essential component of a sustainable future, leading researchers and the public to focus on innovative remediation strategies. As a result, the development of new “green”-oriented, low-cost, and efficient materials for environmental remediation, especially those which facilitate the removal of pollutants from water bodies, remains a vibrant area of research. This Special Issue encompasses 14 original research articles and 2 review papers which aim to explore new methods outside of sorption, biodegradation, and catalytic degradation, while also introducing new materials and composites for effective (waste) water treatment and purification.

In their study, Gao et al. used a biomass, Medulla tetrapanacis (MT), as a sorbent. Known as “da-tong-cao” in China, where it is also famously used as a traditional medicine [1], MT is a well-developed porous 3D structure with an ultra-thin cell wall, predominantly consisting of holocellulose (~82 wt.%) and ash/minerals (~11 wt.%). MT showed high remediation efficiency against methylene blue (MB) and crystal violet (CV), achieving 411 and 553 mg/g adsorption, respectively. Interestingly, the depleted samples were pyrolyzed to biochars, achieving adsorption performances of 320 mg/g for Cu^2+^ and 840 mg/g for Pb2. Crini et al. utilized individualized fibers of a pine wood by-product (Pinus pinaster) were utilized as a sorbent for copper in poly-contaminated aqueous solutions [2]. Upon activation with sodium carbonate, the pine fibers successfully removed 2.5 mg/g copper regardless of the changing pH levels, which ranged from 3 to 5. The presence of the Na^+^ cation at concentrations of 0.1 M did not affect the performance of the material. The adsorption process was rapid, as most of the copper was absorbed within the first 10 min of exposure. The underlying mechanism is considerably more complex due to physisorption, chemisorption, and/or diffusion phenomena. Wang and coworkers followed a multistep protocol to synthesize chitosan modified with pyridine and crosslinked with glutaraldehyde (PYCS) material that achieved 66.2 mg/g of Fe(III) adsorption at pH = 2.5 [3]. The surface pyridine groups were responsible for the formation of stable chelate with Fe(III) ions. PYCS successfully maintained Fe sorption efficiency, since the removal performance only decreased by 29% after six regeneration cycles. Another lignocellulosic biomass composed of Luffa cylindrica fibers was used by Theocharis et al. to synthesize biochar (LCC) after thermal treatment in a nitrogen atmosphere (at 650 °C for 1 h), which was further oxidized (LCC_ox) using HNO_3_ [4]. The materials showed elevated adsorption of U-232 radionuclide at sub-picomolar initial concentrations. Oxidation of the biochar had a positive effect on uranium removal, with the adsorption process being affected by the dispersion pH. The interactions were found to be entropy-driven (ΔH° and ΔS° > 0) based on the creation of inner-sphere complexes. Zhang et al. used recycled agricultural waste from corncobs and KOH/FeCl_3_ to prepare a biochar/alginate composite bead (MCB/ALG) adsorbent via high-temperature pyrolysis (at 800 °C for 2 h) [5]. Although MCB/ALG did not exhibit high porosity (129 m^2^/g), it achieved 1373 mg/g MB removal, and the process was found to be endothermic and spontaneous. This performance was linked to the filling of the pores, electrostatic interactions, and hydrogen bonding. After five cycles of regeneration, the MB removal remained high (85% compared to the first cycle). Tushar Kanti Sen delivered a comprehensive review based on an up-to-date literature overview, focusing on studies that utilized a wide range of solid waste agricultural biomass-based adsorbents (raw, modified, and treated) to purify water that had been polluted with various metal ions [6]. Various important influential physicochemical process parameters, like metal concentration, adsorbent dose, the initial pH of the solution, and the temperature were considered as potential perspectives and conclusions for future work.

Deng and coworkers applied activated carbon in granular form (GAC) for water purification upon the co-presence of three antibiotics, specifically thiamphenicol (THI), sulfamethoxazole (SMZ), and tetracycline (TC) [7]. The carbon granules were obtained from corn stover biomass that was dried, chopped into small pieces, and heated at 500 °C for 2 h and then at 700 °C for 2 h under a N_2_ atmosphere, then underwent further activation via a superheated steam (600 °C, 2.0 MPa, 2 h). GAC had a surface area of 1059 m^2^/g and was predominately microporous (Vmic = 0.488 cm^3^/g). The maximum capacities were around 27, 17, and 30 mg/g for SMZ, TC, and THI, respectively; the sorption was exothermic and spontaneous. The Weber–Morris intraparticle diffusion model and the Boyd kinetic model demonstrated that diffusion across the boundary layer was the primary determinant of the adsorption process. Activated porous carbons (PACs) were studied in another work by Pavlovic et al. as adsorbents of three pharmaceuticals: sulfamethoxazole, trimethoprim, and diclofenac. The removal efficiency was studied using ultra-pure water, humic acid solution, or liquor from real samples collected from wastewater treatment plants [8]. The best removal results were recorded for trimethoprim, followed by diclofenac and sulfamethoxazole. The most notable differentiating factors were linked to the charge and hydrophobicity of the pharmaceuticals at a specific pH. The maximum capacities varied depending on the water matrix; the highest capacity was achieved for sulfamethoxazole and diclofenac in humic acid solution. Multiwalled carbon nanotubes (MWCNTs) were modified through oxidation and acidification [9] using concentrated HNO_3_ and H_2_SO_4_, respectively, and were studied as Mn(II) adsorbents by Dou et al. The chemical treatment had a positive impact on the Mn remediation efficiency, which was almost four times higher than that of the pristine nanotubes. The reliability of the experimental results was thoroughly validated by the PSO-BP simulation, and the findings can serve as a basis for subsequent simulations.

A molecularly imprinted polymer (MIP) was synthesized by Zekker and coworkers through bulk polymerization and utilized in wastewater treatment to enhance the adsorption of specific template molecules [10]. This process utilized ethylene glycol dimethacrylate (EGDMA) as the crosslinker, methacrylic acid (MAA) as the functional monomer, acid black-234 (AB-234) as the template, 2,2′-azobisisobutyronitrile (AIBN) as the initiator, and methanol as the porogenic solvent. The adsorption capacity of the MIP for AB-234 was significantly greater (94%) than that of the NIP (31%) at pH 5, with an estimated maximum capacity of 83 mg/g at 298 K based on the Langmuir model. The MIP exhibited an imprinted factor (IF) of 5.13 and a Kd value of 0.53. Li and coworkers designed a new sulfonation process for an algal/polyethyleneimine (PEI) composite [11]. The functionalized sorbent showed a triple sorption capacity for Sr(II) at pH 4 compared to its non-sulfonated counterpart. The sulfonate groups played a key role in adsorption (as revealed by IR), and sulfonation had a positive impact on thermal stability and porosity. The co-presence of NaCl had a negligible influence on sorption, explaining the good Sr(II) remediation in seawater samples, which was reproducible for at least five cycles. Wilfed and coworkers tested solid-supported ionic liquid consisting of activated silica gel combined with 1-methyl-3-(3-trimethoxysilylpropyl) imidazolium thiosalicylate-based ionic liquid as an extractant of Pb(II) ions from aqueous solutions [12]. The formation of covalent bonds was confirmed by solid-state NMR. The maximum Pb(II) removal capacity was recorded as ~9 mg/g, with the kinetics to be better described by a pseudo-second order model.

Linh Do et al. developed a system that utilizes sulfate-reducing and sulfide-oxidizing processes to treat organic wastewater with high sulfate and sulfide levels [13]. The effects of the COD/SO_4_^2−^ ratio and hydraulic retention time (HRT) on the removal efficiencies of sulfate, COD, sulfide, and electricity generation were examined. The results indicated that the removal efficiencies for COD and sulfate were stable, reaching ~95 and 93%, respectively, throughout the operation. A power density of 18.0 ± 1.6 mW/m^2^ was recorded alongside a sulfide removal efficiency of 93%. However, both sulfide removal efficiency and power density gradually declined after 45 days. Scanning electron microscopy combined with energy-dispersive X-ray analysis revealed that sulfur accumulated on the anode, leading to decreases in sulfide oxidation and electrical generation. This study presents a promising treatment system that could be scaled up for practical applications related to the management of this type of wastewater.

Alhogbi and Balawi presented composite adsorbents consisting of kaolin-derived zeolite and different mass ratios of palm tree fibers (Zeo-FPT) [14]. Mixing the two counterparts resulted in a composite that achieved faster and more efficient removal of methylene blue. The remediation efficiency of the material, which consisted of a one-to-one mass ratio of fibers and zeolite was strong; it achieved >99% removal of a low initial MB concentration in bottled, tap, and well water. He and co-authors prepared a photo-active composite using aminated lignin (AL) and titanate nanotubes [15]. AL was prepared according to the Mannich reaction by modifying technical lignin (TL), while the composite (AL-TiNTs) was formed via a hydrothermal synthesis approach. AL-TiNTs had a specific surface area of 189 m^2^/g, indicating the formation of titanate nanotubes. AL-TiNTs showed an ability to reduce Cr(VI) to Cr(III) and Cr under exposure to visible light, with maximum chromium removal of 90 mg/g. AL-TiNTs also achieved high adsorption performances against Zn^2+^ (64 mg/g), Cd^2+^ (59 mg/g), and Cu^2+^ (66 mg/g), with high efficiency in simulated wastewater for up to four cycles.

Nair and co-authors reviewed the application of biochar-based materials as adsorbents and support for microorganisms to achieve efficient bioremediation of various heavy metal ions and/or pesticides, emphasizing and summarizing the predominant interaction mechanisms and how the immobilized bacteria on biochar contribute to the improvement of bioremediation strategies [16]. In conclusion, this paper outlines the future scopes of this field based on the reviewed and discussed research.
